# The grapefruit: an old wine in a new glass? Metabolic and cardiovascular perspectives

**DOI:** 10.5830/CVJA-2010-012

**Published:** 2010

**Authors:** Peter MO Owira, John AO Ojewole

**Affiliations:** Department of Pharmacology, School of Pharmacy and Pharmacology, Faculty of Health Sciences, University of KwaZulu-Natal, Durban, South Africa; Department of Pharmacology, School of Pharmacy and Pharmacology, Faculty of Health Sciences, University of KwaZulu-Natal, Durban, South Africa

**Keywords:** grapefruit juice, naringin, hesperidin, drug interactions, diabetes mellitus, cardiovascular disease

## Abstract

Grapefruit is a popular, tasty and nutritive fruit enjoyed globally. Biomedical evidence in the last 10 years has, however, shown that consumption of grapefruit or its juice is associated with drug interactions, which, in some cases, have been fatal. Grapefruit-induced drug interactions are unique in that the cytochrome P450 enzyme CYP3A4, which metabolises over 60% of commonly prescribed drugs as well as other drug transporter proteins such as P-glycoprotein and organic cation transporter proteins, which are all expressed in the intestines, are involved. However, the extent to which grapefruit–drug interactions impact on clinical settings has not been fully determined, probably because many cases are not reported.

It has recently emerged that grapefruit, by virtue of its rich flavonoid content, is beneficial in the management of degenerative diseases such as diabetes and cardiovascular disorders. This potentially explosive subject is reviewed here.

## Summary

Grapefruit (*Citrus paradise* Macf., family: Rutacaeae) is popular worldwide, not only because of its taste and nutritive value, but it is also considered to be a functional food that promotes good health.^1^ Scientific evidence backed by molecular biological techniques has shown that grapefruit is most probably a hybrid between pummelo (*C grandis*) and sweet orange (*C sinensis*), followed by introgression back to pummelo.^2^-^4^

The original grapefruit was white-fleshed and very seedy, but other mutated fruit varieties have been selected for either being seedless or increasingly red in colour.^2^ Such varieties include: Duncan/Walters (seedy white), Marsh (seedless, white), Foster (seedy, pink), Thompson (seedless, pink), Redblush (seedless, red), and Ruby, Ray Ruby and Flame (seedless, very red).^2^ These pigmented cultivars have now become more popular and are generally preferred to white grapefruit in the market.^5^

Claims of medicinal properties of grapefruit have led to increased worldwide consumption and renewed interest from basic and clinical research laboratories trying to unravel the ‘mystery’ of this ancient fruit.

## Phytochemistry of the grapefruit

A wide variety of bioactive compounds in grapefruit have been isolated and characterised. Their relative abundance varies according to the variety, geographical location, time of harvesting and the method of processing the grapefruit.^6^ Flavonoids constitute the most abundant bioactive constituents of the grapefruit, and four types of flavonoids (flavanones, flavones, flavonols and anthocynanins) have been identified in the *Citrus* fruits.^7^ Other chemical constituents identified in grapefruit include: limonoid aglycones, glucosides, furanocoumarins (bergamottin, 6′,7′-dihydroxybergamottin), ascorbic acid, folic acid, glucaric acid, carotenoids, pectin and potassium.^8^-^11^

**Fig. 1.**
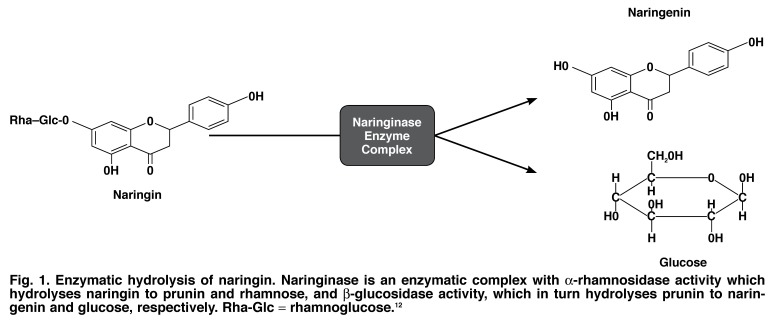
Enzymatic hydrolysis of naringin. Naringinase is an enzymatic complex with α-rhamnosidase activity which hydrolyses naringin to prunin and rhamnose, and β-glucosidase activity, which in turn hydrolyses prunin to naringenin and glucose, respectively. Rha-Glc = rhamnoglucose.^12^

The flavanones (naringin and hesperidin) and limonoids (limonin) are responsible for the bitter taste commonly associated with grapefruit.^12^ Naringin is the most abundant flavanone in grapefruit, but it is converted to its corresponding aglycone (naringenin) and sugars by intestinal bacteria following ingestion^1^,^12^ (Fig. 1). The list of bioactive compounds in grapefruit is by no means exhaustive, and understanding their chemistry in relation to the claimed medicinal benefits is the biggest challenge facing the scientific community.

## A cardiovascular drugs prescriber’s nightmare

A sensational case report published in the *Lancet* last year,^13^ describing a 42-year-old woman who developed venous thrombosis after taking grapefruit for three days while on a contraceptive, ethynylestradiol, marked the return of the ‘dragon’. Grapefruit–drug interactions have been known for nearly a decade now, but unlike drug–drug interactions, food–drug interactions are difficult to legislate. Hence, nothing has been done to address the dangers that patients often expose themselves to while taking grapefruit with prescribed medications.

Cardiovascular drugs constitute more than 50% of the close to 40 or more drugs so far known to interact with grapefruit, and the list is growing.^14^ The accidental observation of pharmacokinetic interaction between ethanol and dihydropyridine calcium channel antagonist (felodipine) when grapefruit juice was used as a flavour to mask the ethanol taste in a study by Bailey *et al.*^15^ opened a Pandora’s box. Other calcium channel blockers that have been discovered to interact with grapefruit include nifedipine, verapamil, diltiazem, nisoldipine, nimlodipine, nitrendipine, amlodipine and nifedipine.^16^-^21^

That the degree of interaction appears to correlate with the oral bioavailability of these drugs, and that the interaction does not affect intravenously administered drugs led researchers to suspect as the main culprit the intestinal cytochrome P450 (CYP3A4), which metabolises these drugs and many others. It has now been established that the bioactive chemical compounds (furanocoumarins: bergamottin and 6′, 7′-dihydroxybergamottin) present in grapefruit inhibit intestinal CYP3A4 activity through a mechanism-based reaction, which causes degradation of the enzyme, hence reducing its levels by as much as 47% within four hours after grapefruit juice ingestion.^1^,^14^,^22^-^24^ It therefore follows that calcium channel blockers, which are extensively metabolised by intestinal CYP3A4, have low oral bioavailability, and are, therefore, most affected by grapefruit inhibition of this enzyme.

Drug interactions with grapefruit juice are not confined to inhibition of only CYP3A4, but also other drug-metabolising cytochrome P450 enzymes such as CYP2C6, CYP2D6, CYP2J2, CYP2C19 and CYP2E1 and many others.^25^-^29^ Drug transporter proteins such as p-glycoprotein (P-gp) and organic anion transporter proteins (OATP) that facilitate drug efflux/influx in the enterocytes have been shown to interact with grapefruit,^30^,^31^ but the specific mechanism of this interaction is still contentious. Both the P-gp and CYP3A4 appear to act synergistically as a barrier to many orally administered drugs.

Cardiovascular drug substrates of P-gp include carvedilol, talinolol, diltiazem, verapamil, simvastatin and lovastatin.^13^,^32^-^35^ However, an interesting observation has been made that despite the fact that digoxin is a strong substrate of P-g, its bioavailability is not affected by grapefruit.^36^ It has since been argued that it is the high oral bioavailability of digoxin (70–80%), rather than lack of grapefruit effect on P-gp, that contributes to its reduced absorption.^37^,^38^

Unlike calcium channel blockers, angiotensin converting enzyme (ACE) inhibitors have not shown any significant interaction with grapefruit juice. However, grapefruit juice has been shown to inhibit the bioactivation of the angiotensin receptor blocker losartan to its active metabolite, thus reducing its efficacy.^39^

Thiazide diuretics and α_1_-adrenergic antagonists, such as doxasozin, terasozin and prazosin, have so far shown no interaction with grapefruit juice.^40^ However, grapefruit juice completely inhibited the conversion of the anti-arrythmic prodrug amiodarone to its active metabolite, N-desthylamiodarone, resulting in 50 and 84% increases in area-under-the-curve (AUC) and maximum plasma concentrations, respectively, of amiodarone. This led to clinical prolongation of QT intervals and torsades de pointes, particularly in patients with pre-existing heart disease or other risk factors, such as hypokalaemia.^14^,^41^

Among the cholesterol-lowering agents, grapefruit is known to increase serum concentrations of simvastatin and its active metabolite, simvastatin acid, and this interaction subsides within three to seven days after ingestion of the last dose of grapefruit juice.^42^,^43^ Similar observations have been made (to a lesser extent though) when other HMG-CoA reductase inhibitors, such as lovastatin and atorvastatin are taken concurrently with grapefruit juice.^14^ However, pravastatin’s (not metabolised in the body) bioavailability was not affected by ingestion of grapefruit juice, indicating that grapefruit juice’s effect on HMG-CoA reductase inhibitors is a consequence of intestinal metabolism of such drugs.^44^ Other cholesterol-lowering agents such as nicotinic acid and common fibric acid derivatives as well as bile acid sequestrants have not been reported to interact with grapefruit juice.^40^

Although grapefruit–drug interactions have been documented in over 225 publications in the scientific literature, involving more than 25 drugs,^45^ the clinical impact on inhibition of intestinal drug metabolism by CYP3A4 has not been fully investigated, and the information available in biomedical literature is largely built on speculations from *in vitro* experiments and a few clinical studies. Evidence gathered so far, therefore, indicates that grapefruit–drug interactions occur when the drug in question is a substrate of CYP3A4, the drug has an inherently low oral bioavailability due to enteric CYP 3A4 metabolism, and when the individual patient expresses sufficient quantities of CYP3A4. Despite the large volume of literature available on this subject, only a few clinical case reports have been documented on grapefruit juice–drug interactions, perhaps because many such cases go unreported.

It is envisaged that grapefruit juice interaction with calcium channel blockers may result in excessive vasodilatation, with symptoms of tachycardia, flushing or hypotension.^46^ However, pronounced decrease in diastolic blood pressure, increase in haemodynamic-related adverse effects, such as increased heart rate and orthostatic hypotension have been reported when felodipine was taken concurrently with grapefruit juice.^47^,^48^

Similarly, concurrent administration of grapefruit juice with HMG-CoA reductase inhibitors, such as atorvastatin, lovastatin or simvastatin at high doses may increase the risk of rhabdomyolysis.^44^,^49^,^50^ With the current trend towards more aggressive lipid-lowering therapy with the statins, the risk of rhabdomylosis is even greater in patients taking grapefruit juice concomitantly.^51^ A possible case of potentiation of the antiplatelet effect of cilostazol by grapefruit juice, leading to purpura has been reported by Taniguchi *et al*.^52^

Liver cirrhosis patients are more dependent on intestinal CYP3A4 for drug metabolism,^1^ and are, therefore, at increased risk. The elderly are particularly vulnerable to grapefruit-induced drug interactions, since they are often on multiple medications, and they experience diminished drug disposition capacity.^48^,^53^,^54^

Genetic polymorphism of the CYP3A4 enzyme would be expected to influence the potential for grapefruit–drug interaction to occur in a patient. Patients who express high levels of intestinal CYP3A4 would extensively metabolise substrate drugs, and hence experience a greater impact of grapefruit juice–drug interactions and *vice versa*. However, no large-scale genotyping data is available for conclusive evidence in this regard.

Positive aspects of grapefruit-induced drug interactions would be related to a potential reduction in costs incurred on reduced treatment regimens of different ailments. Grapefruit contains a number of health-promoting compounds, which may be exploited for therapeutic use. Traditionally, grapefruit–drug interactions have been viewed in terms of enhancement of unwanted adverse effects. But recently, attempts have been made to limit such effects by either modifying the chemistry of the chemical constituents of grapefruit juice, or eliminating them altogether.

Various laboratories have synthesised furanocoumarin dimers, which are believed to be as potent as the natural forms but selective in their inhibition of CYP3A4.^55^,^56^ It is believed that such dimers may be therapeutically exploited to customise grapefruit–drug interactions to specific patients’ needs. A furanocoumarinfree grapefruit juice created by using food-grade solvents and absorption resins failed to inhibit CP3A4 activity and did not increase felodipne’s bioavailability in healthy human volunteers, thus confirming that furanocoumarins are the actual ingredients in grapefruit that enhance felodipine’s bioavailability.^57^

A recent study by Myung et al.^58^ has suggested that autoclaved edible fungi (*Morchella esculenta, Monascus pupureus, Pleuratus sapidus* and *Agarisu bisporus*) bind bergamottin and 6′,7′-dihydroxybergamottin, and can therefore be used to remove furancoumarins from grapefruit juice without affecting its nutritional quality. Previous studies have suggested that heat treatment or UV radiation inactivates bergamottin and 6′,7′-dihydroxybergamottin in grapefruit juice, and therefore eliminates the pharmacokinetic interaction of grapefruit juice with drugs.^59^,^60^ Clinical benefits of such interventions are yet to be seen.

Grapefruit–drug interactions have not, surprisingly, been studied in other organs such as the liver. It is not understood why grapefruit would inhibit intestinal but not hepatic CYP3A4. Is it because the quantities of grapefruit juice used in such clinical studies were not large enough to influence hepatic metabolism? However, our laboratory recently reported a drug interaction of a different kind. We observed that grapefruit juice exacerbates metformin-induced lactic acidosis in rats *in vivo* by facilitating metformin uptake by hepatocytes.^61^ Clearly, the clinical implications of such a finding are significant, given that another biguanide, phenformin was withdrawn from the market when 50% of the patients who took it died due to lactic acidosis.^62^

## Grapefruit and the metabolic syndrome

The metabolic syndrome is a cluster of metabolic abnormalities (currently defined by abdominal obesity, atherogenic dyslipidaemia, raised blood pressure, insulin resistance and or glucose intolerance, pro-inflammatory state and thrombotic state^63^), which increase the risk of developing diabetes and other cardiovascular diseases. Exercise and dietary intake are two of the interventions currently being advocated for among the general public.

Grapefruit has been part of many diets since its incorporation into the ‘Hollywood’ diet of hard-boiled eggs, green vegetables and ‘melba’ toast in 1930 as an anti-obesity ingredient.^64^ A recent study by Fujioka *et al.*^65^ has reported that consumption of whole grapefruit or grapefruit juice is associated with significant weight loss and improved insulin resistance in patients with the metabolic syndrome, compared to placebo. Consumption of grapefruit may, therefore, have beneficial effects in patients with type 2 diabetes mellitus and other degenerative diseases, which may scientifically justify the age-old tradition of dietary supplementation with grapefruit.

Grapefruit consumption has been associated with decreased fasting blood glucose and insulin levels, and serum total cholesterol, low-density lipoprotein and triglyceride levels.^61^,^66^ So much attention has been paid to grapefruit–drug interactions that, to date, the role of grapefruit in prevention of the development of the metabolic syndrome, despite decades of advocacy, is not fully understood.

Dietary flavonoids have been identified as anti-diabetic and may reduce the risk of age-related chronic diseases.^67^ The major flavanones in grapefruit are naringin and hesperidin,^1^,^12^ and many laboratories have attempted to probe whether these flavonoids may be linked to the reduced risk of degenerative diseases associated with grapefruit consumption. Naringin, like insulin, has been shown to decrease microsomal triglyceride transfer protein [necessary for hepatocyte assembly and secretion of apolipoprotein (apo)B-containing lipoproteins which confer an increased atherosclerotic risk] expression *in vitro*.^68^ This therefore suggests that naringin may be useful in activating insulinsignalling pathways important for the regulation of hepatocyte lipid metabolism.

Combined treatment with naringin and vitamin C has been demonstrated to ameliorate streptozotocin-induced diabetes in rats.^69^ Jung *et al.*^70^,^71^ have reported that hesperidin and naringin are beneficial for improving hyperlipidaemia and hyperglycaemia in type 2 diabetic animal models by partly regulating fatty acid and cholesterol metabolism and affecting gene expression of glucose-regulating enzymes. Preliminary results from our laboratory also indicate that grapefruit juice regulates the activities of hepatic glucose-6-phosphatase and phosphoenolpyruvate carboxykinase, respectively, in rats.^72^

All these results vindicate flavonoids in the anti-diabetic effects of grapefruit. However, it is still not clear whether the apparent inhibitory effects of these flavonoids on the hepatic enzymes controlling glucose homeostasis act directly or indirectly via upstream regulators of these enzymes, such as adenosine monophosphate protein kinase (AMPK), the ‘master energy sensor’, which is known to regulate the activities of the enzymes.^73^-^75^ That these flavonoids or their derivatives may be the ultimate anti-diabetic agents in grapefruit is a speculative possibility, given that AMPK modulators are currently being investigated as potential anti-diabetic agents.^76^

## Grapefruit and cardiovascular diseases

That *Citrus* flavonoids are promising compounds against cardiovascular diseases is a dream becoming reality. Epidemiological studies are unanimous that increased dietary intake of flavonoids has been associated with reduced risk of ischaemic stroke and cardiovascular diseases.^77^,^78^ The protective effects of flavonoids include: anti-ischaemic, antioxidant, vasorelaxant and antithrombotic properties.

It has been suggested that flavonoids decrease the risk of coronary heart diseases by improving coronary vasodilatation, decreasing the ability of the platelets to clot, and preventing oxidation of low-density lipoproteins (LDL).^79^ Recent studies have shown that naringenin inhibits secretion of apoB and enhances LDL receptor-mediated apoB uptake.^80^ Hesperidin has similarly been reported to increase high-density lipoprotein (HDL) and lower LDL, plasma triglycerides and total lipids in rats.^81^

Hesperidin and naringenin have been reported to cause vasorelaxation of rat intact aortic rings by inhibition of different phosphodiesterase isoenzymes.^82^,^83^ Another study by Yamamoto *et al*.^84^ also reported that glucosyl-hesperidin lowers blood pressure in spontaneously hypertensive rats (SHR) and prevents endothelial dysfunction and oxidative stress in SHR.^85^ A recent study by Rajadurai *et al*.^86^ has demonstrated that naringin prevents mitochondrial dysfunction during isoproterenol (ISO)-induced myocardial infarction in rats, suggesting that naringin has a cardioprotective role against myocardial infarction, perhaps due to its reported antioxidant effects.^87^

However, grapefruit has been implicated in the prolongation of QTc in healthy volunteers,^88^ as well as in patients with postischaemic dilated cardiomyopathy or hypertensive cardiomyopathy.^89^ Naringenin has been identified as the culprit.^90^ This observation suggests that naringenin in grapefruit has pro-arrythmic actions which may block the therapeutic effects of anti-arrythmic drugs.

These observations, therefore, strongly suggest that hesperidine and naringin, acting alone or synergistically with other chemical compounds in grapefruit, affect the cardiovascular system in many ways. However, it is not clear at this stage how these flavonoids could be mediating such effects. It is tempting to speculate that the actions of hesperidine and naringin (or naringenin) could be mediated by AMPK, which is known to increase glucose uptake, fatty acid uptake and utilisation, and glycolysis in the heart and other peripheral tissues.

It is therefore not surprising that the cardioprotective effects of metformin have now been recognised, and AMPK-mediated pathways are currently considered potential therapeutic targets in cardio-metabolic diseases. It has recently been reported that a single dose of metformin results in acute increase in AMPK activity, and induces a significant reduction in infarct size 24 hours after metformin administration.^91^ Metformin-like effects of grapefruit juice in the regulation of blood glucose have been reported.^61^ Could grapefruit have similar effects on the cardiovascular system?

## Conclusions

In the last 10 years, grapefruit has been a pharmacologoist’s nightmare, given its popularity and potential for interaction with many therapeutic drugs. To date, no clear guidelines have been put forward to protect vulnerable patients against the hazardous consequences of grapefruit–drug interactions. However, it is now emerging that apart from drug interactions, which have largely been attributed to furanocoumarins, flavonoids such as naringin and hesperidin could be playing more important roles in the prevention of diabetes and cardiovascular diseases. Attention has now shifted to investigating the molecular mechanisms by which these flavonoids exert their protective cardiovascular effects. In the coming years, basic and clinical research in cardiovascular pharmacology should be focused on grapefruit and its flavonoids and/or their chemical derivatives.
